# Studies on an alkali-thermostable xylanase from *Aspergillus fumigatus* MA28

**DOI:** 10.1007/s13205-011-0020-x

**Published:** 2011-08-13

**Authors:** Bijender Kumar Bajaj, Massarat Abbass

**Affiliations:** 1 School of Biotechnology, University of Jammu, Jammu, 180 006 India; 2 Present Address: Biotechnology and Fermentation Group, Department of Animal Sciences, Gerlaugh Hall, Ohio Agricultural Research and Development Centre (OARDC), The Ohio State University, 1680 Madison Avenue, Wooster, OH 44691 USA

**Keywords:** Xylanase, Alkali-thermostable, *Aspergillus fumigatus*, Production, Agro-residues, Purification

## Abstract

An alkalitolerant fungus, *Aspergillus fumigatus* strain MA28 produced significant amounts of cellulase-free xylanase when grown on a variety of agro-wastes. Wheat bran as the sole carbon source supported higher xylanase production (8,450 U/L) than xylan (7,500 U/L). Soybean meal was observed to be the best nitrogen source for xylanase production (9,000 U/L). Optimum medium pH for xylanase production was 8 (9,800 U/L), though, significant quantities of the enzyme was also produced at pH 7 (8,500 U/L), 9 (8,200 U/L) and 10 (4,600 U/L). The xylanase was purified by ammonium sulphate precipitation and carboxymethyl cellulose chromatography, and was found to have a molecular weight of 14.4 kDa with a *V*_max_ of 980 μmol/min/mg of protein and a *K*_m_ of approximately 4.9 mg/mL. The optimum temperature and pH for enzyme activity was 50 °C and pH 8, respectively. However, the enzyme also showed substantial residual activity at 60–70 °C (53–75%) and at alkaline pH 8–9 (56–88%).

## Introduction

Xylan, consisting of a backbone of β-(1 *→* 4) linked d-xylopyranosyl units with various substitutions, is the second most abundant biopolymer in plant cell walls after cellulose, and represents the major polysaccharide of hemicelluloses. Endo-β-1,4-xylanase (EC 3.2.1.8) and β-xylosidase (EC 3.2.1.37) are the key enzymes involved in the hydrolysis of the xylan backbone although some other enzymes are required for debranching (Kuhad and Singh 1993). Xylanases have high potential for biotechnological applications. For instance, xylanases can be used to improve the digestibility and nutritional value of ruminant fodder, to facilitate composting process, to improve the quality of bread, to develop environmentally friendly pulp bleaching process and to transform lignocellulosic materials to fermentable products (Kuhad and Singh 1993; Beg et al. 2001; Maalej et al. 2009; Singh et al. 2011). In pulp and paper industry, xylanase-treatment of kraft pulp reduces the dependence on the use of hazardous chemicals required for bleaching but in this case xylanases must be free from any cellulase activity (Kuhad and Singh 1993; Beg et al. 2001; Singh et al. 2011).

For bulk production of industrial enzymes, the cost of the substrate constitutes one of the most important factors in determining the overall economy of the process. Agriculture-based wastes such as wheat bran, sawdust, rice straw, corncob, wood husk and maize bran are excellent low-cost substrates for xylanase production (Bajaj and Singh 2010; Murthy and Naidu 2010); India, being an agriculture-based economy, generates huge quantum of agro-residues which are difficult to dispose off, and their use as substrates for xylanase production will not only combat environmental pollution but will also reduce the cost of enzyme production.

Xylanases are produced by many bacterial and fungal species but most of the enzymes characterized to date are optimally active only at acidic, neutral or slightly alkaline pH, and at temperature between 40 and 60 °C. These characteristics fall short of the required criteria for industrial applications and to fulfil the requirement of more robust xylanases, recently more thermostable and alkalistable xylanases have been reported (Sharma and Bajaj 2005; Sudan and Bajaj 2007; Nair et al. 2008; Bajaj and Singh 2010; Murthy and Naidu 2010; Singh et al. 2011). Fungi have been studied widely for thermo- and alkali-stable xylanases over bacteria as they offer many advantages—fungi are biodiverse, produce extracellular enzymes which obviates the need for cell breakage, can efficiently utilize complex agro-industrial wastes as substrates for enzyme production, produce higher amounts of enzymes, are more tolerant and better adapted to changes in the environmental factors for growth and produce several auxiliary enzymes which are important in debranching of substituted xylans (Bakri et al. 2010; Haltrich et al. 1996; Sudan and Bajaj 2007; Bakri et al. 2010; Pal and Khanum 2010; Shuvaeva and Sysoeva 2010).

We report here our study of a thermo- and alkali-stable xylanase from *Aspergillus fumigatus* MA28.

## Materials and methods

### Chemicals, media and medium components

All the chemicals, media, and media components used in this study were procured from HiMedia Laboratories Ltd., Ranbaxy Fine Chemicals Ltd., Qualigens Fine Chemicals Ltd. (India) and Sigma Chemicals Ltd. and Merck and Co. Inc. (USA).

### Isolation of xylanolytic fungi

Samples of alkaline soil, soil samples beneath heaps of rice and wheat straw or sugarcane bagasse, cow dung manure, poultry waste, sawdust, among others, were collected from alkaline, hot and humid locations in the vicinity of decaying organic matter, and used for the isolation of xylanolytic fungi (Sudan and Bajaj 2007). Samples were suspended in saline (0.85% NaCl) and suitable dilutions were spread-plated on potato-dextrose-agar (PDA) and plates were incubated at 30 °C. Fungal colonies which appeared after 3–5 days of incubation at 30 °C were transferred from PDA to xylan agar medium. Xylan agar medium contained (g/L): ammonium sulphate 3, potassium dihydrogen phosphate 3, ammonium acetate 6, oat spelt xylan 5, agar 20; pH 8–10). Plates were incubated at 30 °C for 3–5 days and colonies developed were assayed for their xylanase producing ability by Congo red staining (Sharma and Bajaj 2005). A total of 40 fungal isolates were examined for xylanolytic activity. Selected isolates were examined further for xylanase producing ability under submerged fermentation (pH 8–9). All fungal isolates were maintained on PDA slants at 4 °C.

### Growth, and enzyme production and assay from selected isolates

Selected xylanolytic fungal isolates were grown on PDA for 5 days and inoculated (3 discs of 3 mm each/100 mL) into enzyme production medium containing (g/L): ammonium sulphate 3, potassium dihydrogen phosphate 3, ammonium acetate 6, xylan 5, KCl 0.5, magnesium sulphate 0.5, ferrous sulphate 0.1; pH 8) and fermentation was conducted in shaking-incubator (Innova, New Brunswick, USA) at 30 °C (180 rpm). After suitable intervals of time, samples were withdrawn and centrifuged at 10,000×*g* for 10 min at 4 °C (Sigma 3K30, UK). The pellet was dried to constant weight in a hot air oven maintained at 90 °C to determine the amount of biomass, and supernatant was assayed for xylanase, carboxymethyl cellulase (CMCase) and FPase (filter paper activity) activities. Xylanase activity was assayed by using xylan (0.5% w/v in Tris buffer, 50 mM, pH 8) as the substrate at 45 °C. The reducing sugars released were assayed by dinitrosalicylic acid (DNSA) method using xylose as standard (Miller 1959). One unit of xylanase activity (IU) is defined as the enzyme necessary to release 1 μmol of reducing sugar or xylose equivalent per min under assay conditions. CMCase and FPase were also assayed by employing DNSA method using carboxymethyl cellulose (CM-cellulose) and filter paper as substrates, respectively (Sudan and Bajaj 2007). One unit (IU) of CMCase or FPase was defined as the amount of enzyme required to release 1 μmol of reducing sugar equivalent per min under assay conditions. Qualitative analysis of xylanolytic activity was done by using well plate assay. For this, the enzyme (70 μl) was pipetted into wells of xylan agar plates. The wells were made using a 6 mm cork borer. The plates were incubated overnight at 30 °C and then stained with Congo red. Zones of clearance were suggestive of the presence of xylanase. Isolate MA28 which yielded maximum xylanase activity was selected for further studies.

Identification of the isolate MA28 was done by culture morphological and microscopic examination (Raper and Fennell 1965), and by internal transcribed spacer (ITS) sequence analysis. The ITS region of fungal rDNA which includes ITS 1, 5.8S and ITS 2 regions was amplified by using universal ITS region primers (ITS 1, 5′-TCC GTA GGT GAA CCTGCG G-3′, and ITS 4, 5′-TCC TCC GCT TAT TGA TAT GC-3′) (Bakri et al. 2010). The amplified DNA was sequenced directly (ABI Biosystems) and the sequence was aligned with the consensus fungal ITS sequences available in the GenBank (NCBI) using Mega BLAST. Multiple ITS sequence alignment was performed using Clustal W 2. The sequence was submitted to GenBank (NCBI) and accession number obtained (*JN112377*).

### Xylanase production with different carbon and nitrogen sources

To test the effect of different carbon sources on xylanase production, xylan of the production medium was replaced with either of the different agro-based substrates (10 g/L) namely, wheat bran, rice bran, sawdust, rice straw, powdered corncob, wood husk, maize bran, wheat flour mill waste or cotton seed cake as the sole carbon source. Prior to use, these substrates were crushed, sieved (mesh size 20), and steam hydrolyzed by autoclaving at 15 psi for 15 min. To examine the effect of different nitrogen sources on xylanase production, the production media containing xylan (5 g/L) was used with either of the following nitrogen sources (5 g/L): yeast extract, urea, soybean meal, ammonium sulphate, peptone, gelatin, tryptone, beef extract, potassium nitrate or ammonium nitrate. Five-day old fungal cultures grown on PDA were used to seed the media containing different carbon and nitrogen sources followed by incubation at 30 °C with shaking (180 rpm). Samples withdrawn after various time intervals were assayed for activity of xylanase, CMCase and FPase.

### Effect of initial medium pH on xylanase production

Production medium containing xylan (5 g/L) as the carbon source and soybean meal (5 g/L) as the nitrogen source pre-adjusted to pH between 5 and 10 with NaOH (0.1 M) or HCl (0.1 M), was used to determine the effect of initial medium pH on xylanase production. Growth was initiated by seeding the medium with a 5-day-old PDA grown culture followed by incubation at 30 °C with shaking (180 rpm).

### Xylanase purification

Freshly developed enzyme was subjected to ammonium sulphate (AS) precipitation at different saturation levels (20–100%). Each fraction was tested for protein content and xylanase activity. The fractions that had substantial xylanase activity were pooled, and dialyzed against phosphate buffer (pH 7) for 12 h. Buffer was replaced with fresh one after every 4 h during dialysis. Dialyzed preparation was used for ion exchange chromatography. The CM-cellulose column (3.5 × 50 cm) was equilibrated with 10 volumes of the equilibration buffer (Tris buffer, 10 mM, pH 9), and the sample was loaded. Elution was carried out by using increasing concentrations of NaCl (0.25–1.0 M), and fractions collected were analysed for xylanase activity and protein content. Protein content in the samples was determined by the method of Lowry et al. (1951). The fraction with substantial activity was examined by Native-polyacrylamide gel electrophoresis (PAGE) for zymogram study, and by sodium dodecyl sulphate-PAGE (SDS-PAGE) for molecular weight determination (Sambrook et al. 1989). Acrylamide concentration in resolving gel and stacking gel was 12 and 5%, respectively, and electrophoresis was performed at 100 V for 4 h. Purified enzyme was loaded in duplicate on native gel, and after electrophoresis, the gel was cut vertically into two equal halves. For zymogram analysis, one half of the native gel was overlayered on agar plate containing agar (1%, w/v) and xylan (0.5%, w/v), and incubated at 50 °C for 1–2 h, followed by Congo red staining of agar plate and observation for zone of clearance which is suggestive for positive zymogram reaction; and the other half of native gel was stained with coomassie brilliant blue (CBB) and observed for protein bands after destaining.

### Characterization of purified xylanase for some properties

Kinetic parameters (*K*_m_ and *V*_max_) of purified xylanase were examined by studying the reaction rate at various substrate (xylan) concentrations (5–40 mg/mL). Substrate concentration was plotted against the reaction rate to determine whether the enzyme obeys Michaelis–Menten kinetics, and *K*_m_ and *V*_max_ were determined from the Lineweaver–Burk plot.

For studying the effect of temperature on xylanase activity, the enzyme assay was conducted at different temperatures (30–100 °C). The thermostability of the enzyme was examined by pre-incubating the purified enzyme (without substrate) at 60–100 °C for different intervals of time, and then assaying the residual activity.

To study the effect of pH on xylanase activity, the substrate used in enzyme assay was prepared in different buffers (10 mM) such as citrate buffer (pH 3, 4, 5 and 6), tris buffer (pH 7, 8 and 9) and glycine-NaOH buffer (pH 10). For determining pH stability, the enzyme (without substrate) was pre-incubated at different pH (6–10) using appropriate buffers, for varying time periods, and then assayed for the residual activity.

To determine the effect of various ions/additives on xylanase activity, either of the ion/additive such as FeSO_4_·7H_2_O, NH_4_Cl, ZnSO_4_·7H_2_O, MnSO_4_·H_2_O, CaCl_2_·2H_2_O, MgSO_4_·7H_2_O, SDS or ethylenediaminetetraacetic acid (EDTA) was included individually in the enzyme assay reaction mixture at final concentration of 10 mM, and activity assay was conducted.

All the analytical experiments were set up in triplicates and data presented is the mean of three different experiments.

## Results and discussion

### Xylanolytic organism, growth profile and time-course of xylanase production

Out of 40 fungal isolates screened, MA28 (the isolate from soil beneath big heap of rice husk from a rice mill) was selected for detailed studies as it showed the highest xylanase activity qualitatively as well as under submerged fermentation (at alkaline pH). The identity of the isolate was established on the basis of macro (colony, growth, exudates, reverse colour, etc.) and micro (heads, condiphores, phialides, vesicles, conidia etc.) morphological characteristics (Raper and Fennell 1965). Fungal colonies on xylan agar, and on Czapek’s Yeast Autolysate agar were fast spreading, powdery, initially white turning castor-grey to grayish-green later with the development of conidial heads, and reverse was faintly pale. Conidial heads were mostly columnar, conidiophores were smooth, with 300 μm length and 4.8–8.0 μm wide, gradually enlarging upward and ending into the apical flask shaped vesicles (diameter 19.2–32.0 μm), usually fertile on three quarters, uniseriate, phialides 6.4–8.0 to 1.6–3.2 μm; conidia were bluish green, globose to subglobose, echinulate, mostly 2.4–3.2 μm in diameter, sclerotia or cleistothecia were absent. Based on the characteristics, the fungal strain was identified as *Aspergillus fumigatus*. To confirm the identity of the organism further, ITS sequence (ITS 1 region, 5.8S rRNA gene and ITS 2 region) was amplified (593 base pair sequence) and compared with that available in GenBank data base using Mega BLAST (NCBI). The obtained sequence showed 100% homology with available sequence of *A. fumigatus* (AF176662.1) in GenBank database. The sequence has been deposited in GenBank under accession number *JN112377*. The isolate MA28 was designated as *A. fumigatus* MA28. Recently, various fungal isolates have been reported to be excellent xylanase producers (Sudan and Bajaj 2007; Fang et al. 2010; Antoine et al. 2010; Shi et al. 2011; Bajaj et al. 2011). A thermostable xylanase from a newly isolated thermophilic fungus *Talaromycesthermophilus* was purified and characterized (Maalej et al. 2009).

**Fig. 1:**
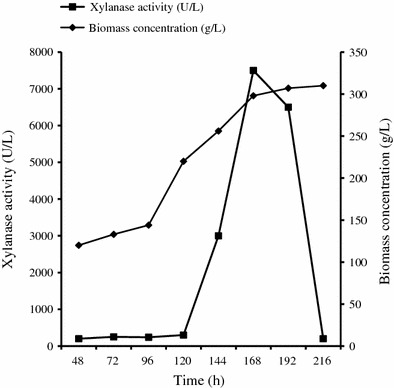
Growth and time-course of xylanase production by *Aspergillus fumigatus* MA28. The organism was cultivated under shaking (180 rpm) at 30 °C. Amount of biomass was determined on dry weight basis (in hot air oven at 90 °C), and xylanase activity was assayed by determining the ability of enzyme to cause release of reducing sugars from xylan

The time-course of growth and xylanase production by *A.fumigatus* MA28 showed that for initial 120 h growth was slow and there was no xylanase production. Xylanase production commenced after 144 h (3,000 U/L) and increased steeply and reached to the maximum level after 168 h (7,500 U/L). After 192 h, xylanase activity suffered a slight decrease (6,500 U/L), however, afterwards xylanase activity decreased sharply (Fig. 1). The abrupt decrease in xylanase activity may be attributed to the activation of certain proteases or to the interaction of xylanase with some other medium/cell-secreted components or due to the inhibition of the enzyme by the end products (Peixoto-Nogueira et al. 2009). Growth and product formation share quite complex relationship, at times they go-parallel while sometimes show partial-association (Nizamudeen and Bajaj 2009). Furthermore, optimal time for maximum enzyme production varies from organism to organism. *Penicillium canescens* showed maximum xylanase production after 192 h of incubation (Antoine et al. 2010) while *Fusarium solani* F7 (Gupta et al. 2009) and *Aspergillus sydowii* SBS 45 (Nair et al. 2008) did so after 6 and 9 days of fermentation, respectively. *Aspergillus niveus* RS2 yielded maximum xylanase after 5 days of fermentation (Sudan and Bajaj 2007). Thus, it may be understood that time for maximum enzyme production depends on the type of the microbial strain, cultural and environmental conditions and genetic make-up/or potential of the organism (Peixoto-Nogueira et al. 2009; Bajaj and Singh 2010). Furthermore, the enzyme preparation altogether lacked CMCase and FPase activities. It is pertinent to mention here that when the intended application of the xylanase is in the pulp and paper industry, the enzyme must be free of cellulolytic activity else the cellulose fibres may be damaged. There are many reports where-in cellulase-free xylanases have been reported from fungi (Dutta et al. 2007). However, negligible or significant contamination of fungal xylanases with cellulases has also been reported (Ruckmanl and Rajendran 2001; Sudan and Bajaj 2007).

### Xylanase production using various carbon sources

**Fig. 2:**
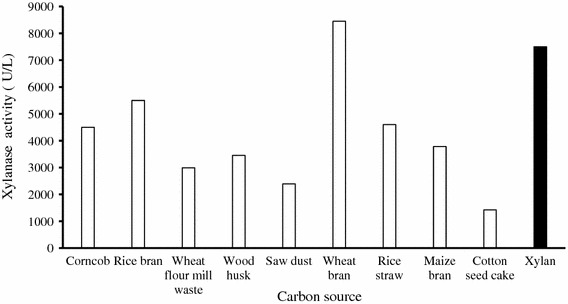
Xylanase production from various agriculture-based carbon sources. Fermentation was executed under shaking (180 rpm) at 30 °C. *Black bar* indicates xylanase production when xylan was used as carbon source (5 g/L), while the *colourless bars* show xylanase production when xylan was replaced with either of the agriculture-based carbon sources (10 g/L)

The cost of the substrate plays a critical role in determining the overall economics of enzyme production. Low-value agriculture-based raw materials have been extensively explored as substrates for microbial enzyme production (Bajaj and Singh 2010; Pal and Khanum 2010; Bajaj and Sharma 2011; Bajaj et al. 2011). *A.fumigatus* MA28 showed varying level of cellulase-free xylanase production on all the agro-based substrates examined in this study (Fig. 2). Wheat bran was found to be the best inducer of xylanase and remarkably performed even better than pure xylan (activity: 8,450 U/L on wheat bran vs. 7,500 U/L on xylan), and was followed by rice bran (5,500 U/L), rice straw (4,600 U/L) and corn cobs (4,500 U/L). Thus, among the agro-wastes, wheat bran holds the greatest promise for cost-effective production of the xylanase besides it may also provide a substantial quantity of soluble arabinoxylans. Reports in literature suggest that pure xylan can be an excellent carbon source, which not only results in increased yield of xylanase but often causes a selective induction of xylanase with no or little formed cellulase (Sudan and Bajaj 2007). Wheat bran has been used as substrate for xylanase production from various organisms including *A. niveus* RS2 (Sudan and Bajaj 2007), *A. sydowii* SBS 45 (Nair et al. 2008), *A. niger* DFR-5 (Pal and Khanum 2010), *Rhizopus* var. *microsporus* 595 (Shuvaeva and Sysoeva 2010) and *Streptomyces* sp. 7b (Bajaj and Singh 2010). Evaluation of xylanase production by *A. fumigatus* RP04 and *A. niveus* RP05 showed that the former produced high level of xylanase on agricultural residues (corncob or wheat bran), whereas latter produced more xylanase on birchwood xylan (Peixoto-Nogueira et al. 2009**)**. Besides wheat bran, other agro-industrial residues like soy meal, wheat and rice straw, rice bran, wood husk, coba husk, lignocellulosic coffee by-products, corn steep liquor etc. have been employed as substrates for xylanase production from different fungi (Gupta et al. 2009; Antoine et al. 2010; Fang et al. 2010; Murthy and Naidu 2010). Thus, there is an intense focus on the valorization of agro-industrial residues for production of value-added products.

### Xylanase production using various nitrogen sources

**Fig. 3:**
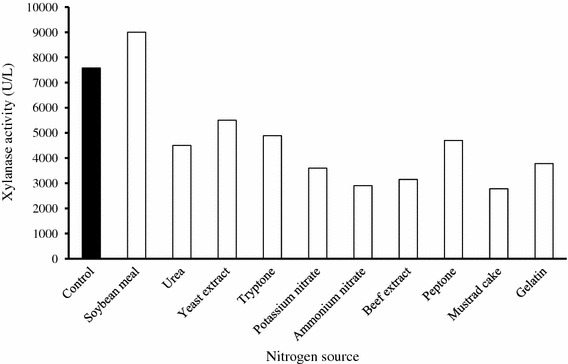
Xylanase production by *Aspergillus fumigatus* MA28 using various nitrogen sources. Fermentation was executed under shaking (180 rpm) at 30 °C. *Black bar* indicates xylanase production when ammonium salts (ammonium sulphate and ammonium acetate) were used as nitrogen source while the *colourless bars* show when ammonium salts were replaced with various nitrogen sources (5 g/L)

**Fig. 4:**
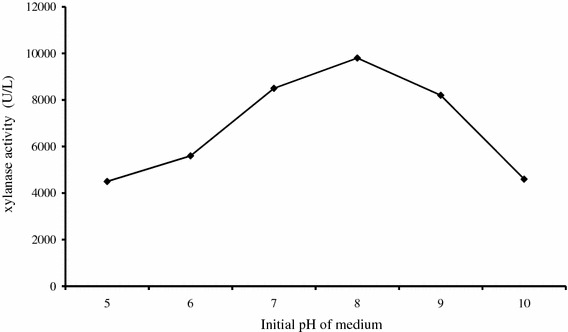
Effect of initial pH of medium on xylanase production by *A. fumigatus* MA28. The initial pH of the production medium was adjusted at pH 5–10 using either HCl (0.1 M) or NaOH (0.1 M), and fermentation was carried out under shaking (180 rpm) at 30 °C

The mechanisms that govern the formation of extracellular enzymes are influenced by the availability and type of nitrogenous precursors for protein synthesis. Nitrogen source can significantly affect the pH of the medium during the course of fermentation which in turn may influence enzyme activity and stability. In the present study, soybean meal supported higher enzyme production (9,000 U/L) as compared to the control (7,580 U/L), while all other nitrogen sources led to reduced xylanase production (Fig. 3). Soybean meal is complex and conditioned nitrogen source and does not cause catabolite repression (Sharma and Bajaj 2005). Probably this organic nitrogen source contains majorly all kinds of amino acids which can be readily absorbed by fungal mycelia. Mustard cake is quite rarely used as a nitrogen source for enzyme production (Bajaj and Sharma 2011); in the present study it did support xylanase production though to a lesser extent (Fig. 4). Similar to our results, *A. sydowii* SBS 45 (Nair et al. 2008) showed maximum xylanase production when soybean meal was employed as nitrogen source. However, *A. niveus* RS2 produced maximum xylanase when yeast extract was used as nitrogen source (Sudan and Bajaj, 2007). *Penicillium canescens* (Antoine et al. 2010) preferred casein, peptone and soybean meal as nitrogen sources for maximum xylanase production while *Penicillium* sp. CFR 303 (Murthy and Naidu 2010) performed best with peptone as nitrogen source.

### Effect of initial medium pH on xylanase production

Medium pH has got profound influence on the organism’s growth as well as on its ability for enzyme production. Unfavourable pH may not allow adequate growth and can also influence the activity and stability of enzyme. Fermentation was carried out in production medium at different pH values (5–10). Though, maximum xylanase production was observed in medium with pH 8 (9,800 U/L), substantial xylanase titre was observed at pH 9 (8,200 U/L) and pH 10 also (4,600 U/L) (Fig. 4). Majority of researchers have reported acidic pH (5–6.5) as the most appropriate for maximum enzyme production from fungi (Gupta et al. 2009; Murthy and Naidu 2010). However, *A. niveus* RS2 (Sudan and Bajaj 2007), *A. flavus* (Ruckmanl and Rajendran 2001) and *A. sydowii* SBS 45 (Nair et al. 2008) exhibited maximum production of xylanase at pH 8, 9 and 10, respectively. In the present investigation, *A. fumigatus* MA28 has showed sufficient alkalitolerance and capability to grow and produce enzyme at alkaline pH.

### Purification of xylanase

**Fig. 5:**
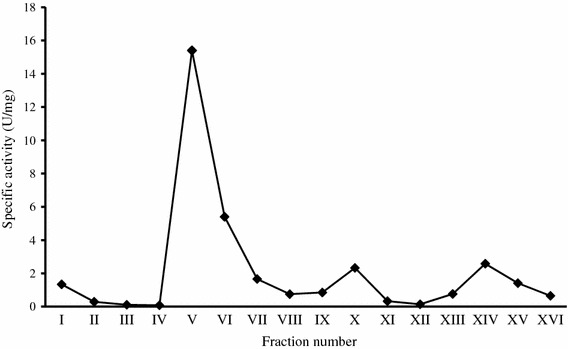
Specific activity of xylanase in different fractions eluted from carboxymethyl cellulose chromatography column. Elution was done by using increasing concentration of NaCl (0.25–1 M) solution

Variety of approaches such as salt precipitation, ion exchange chromatography, gel filtration chromatography etc. have been employed for purification of enzymes (Nair et al. 2008; Gupta et al. 2009; Bajaj and Singh 2010; Bajaj and Sharma 2011). In the present study, AS precipitation of xylanase showed maximum activity at 20% saturation, and led to purification of xylanase by 7.95-fold (Table 1). CM-cellulose chromatography (3.5 × 50 cm) further increased the purification of xylanase (fraction V) by 38.5-fold (Fig. 5; Table 1). Native-PAGE analysis of fraction V from CM-cellulose chromatography showed the presence of a single band on the stained half of duplicate native gel while the unstained half showed positive zymogram reaction (appearance of zone of clearance on xylan agar plate after Congo red staining). SDS-PAGE analysis showed the presence of single band of molecular weight of approximately 14.4 kDa. Similar to the present study, xylanase from *A. niveus* RS2 was purified by AS precipitation and CM-sephdex chromatography by 5.08-fold, and the enzyme had a molecular weight of 22.5 kDa (Sudan and Bajaj 2007). *A. sydowii* SBS 45 xylanase was purified by 93.41-times by using AS precipitation, gel filtration and anion exchange chromatography. Antoine et al*.* (2010) purified xylanase from *P. canescens* by 1.39-fold using AS precipitation. Purified xylanase from *F. solani* F7 yielded a single band with a molecular weight of 89 kDa (Gupta et al. 2009).

**Table 1:** Purification of xylanase from *Aspergillus fumigatus* MA-28

Purification step	Fraction volume (mL)	Total protein (mg)	Activity (U)	Specific activity (U/mg protein)	Purification fold	Recovery (%)
Crude enzyme	500	875	350	0.4	1	100
Ammonium sulphate precipitation	8	16.4	52.24	3.18	7.95	14.92
Carboxymethyl cellulose chromatography	4	2.0	30.8	15.4	38.5	8.8

All the purification steps were executed at 4 °C

### Kinetic parameters

Analysis of kinetic parameter of purified xylanase from *A. fumigatus* MA28 showed that it had *K*_m_ of 4.9 mg/mL and *V*_max_ of 980 μmol/min/mg protein. Nair et al. (2008) studied the kinetic parameters of two xylanases from *A. sydowii* SBS 45 and reported that *K*_m_ value of xylanase I for birch wood xylan was 3.18 mg/mL and for oat spelt xylan 6.45 mg/mL, while the *K*_m_ value of xylanase II for birch wood xylan was 6.51 mg/mL and for oat spelt xylan 7.69 mg/mL. *V*_max_ of xylanase I and II for birch wood xylan was 1,191 and 1,587 μmol/min/mg protein, respectively. *V*_max_ of xylanase I and II for oat spelt xylan was 2,604 and 2,381 μmol/min/mg protein, respectively. *Penicillium* sp. CFR 303 showed *K*_m_ of 5.6 mg/mL, and *V*_max_ of 925 μmol/min/mg with birchwood xylan as a substrate (Murthy and Naidu 2010). The xylanase from *A. niveus* RS2 had *K*_m_ and *V*_max_ values of 2.5 mg/mL and 26 μmol/mg/min, respectively (Sudan and Bajaj 2007).

### Effect of temperature on xylanase activity

**Fig. 6:**
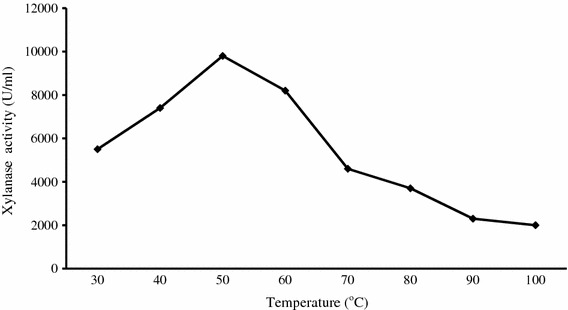
Effect of temperature on activity of *A. fumigatus* MA28 xylanase. Activity assay was conducted at different temperatures (30–100 °C) by incubating the enzyme assay mixture in water bath set at appropriate temperature

Activity assay at different temperatures (30–100 °C) indicated that *A. fumigatus* MA28 xylanase exercises maximum activity at 50 °C (9,800 U/L), however, significant activity was observed at 60 °C (8,200 U/L) and 70 °C (4,600 U/L) also (Fig. 6). However, at still higher temperatures (70–100 °C) reduction in enzyme activity occurred. Several fungal xylanases have been reported to show optimum activity at 50 °C (Srinivasan and Rele 1999; Dutta et al. 2007; Nair et al. 2008; Murthy and Naidu 2010). However, xylanases from *A. tubingensis* and *A. terreus* showed optimum activity at 65 °C (Bakri et al. 2010), and those from *A. fumigatus* and *A. niveus* displayed optima at 60–70 °C (Peixoto-Nogueira et al. 2009). Similarly, Maalej et al. (2009) reported temperature optima of 75–80 °C for *T.thermophilus* xylanase.

### Effect of pH on enzyme activity

**Fig. 7:**
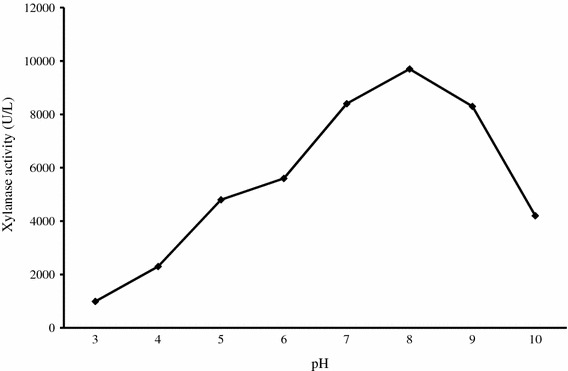
Effect of pH on activity of *A. fumigatus* MA28 xylanase. The activity assay was conducted at different pH (3–10) using appropriate buffers. The substrate solution used in the enzyme assay mixture was prepared either in citrate buffer (pH 3, 4, 5 and 6), tris buffer (pH 7, 8 and 9) or glycine-NaOH (pH 10) to expose the enzyme to different pH

Activity of the purified xylanase was assayed at different pH (3–10) by using appropriate buffers. It was remarkable to observe that enzyme activity increased continuously with increase in pH from 3 to 8 and reached maximum at pH 8 (9,700 U/L) as shown in Fig. 7. Nonetheless significant enzyme activity was observed at pH 9 (8,300 U/L) and pH 10 (4,200 U/L). Results indicate very alkaliphilic feature of enzyme. It is worth mentioning here that *A.fumigatus* MA28 xylanase has major levels of alkalistability and thus, could be of potential importance for pulp bleaching applications. Generally, microbial xylanases show activity in acidic or neutral range. The pH optima of the xylanases from *A. fumigatus* and *A. niveus* (Peixoto-Nogueira et al. 2009**)**, from *F. solani* F7 (Gupta et al. 2009) and from *Penicillium* sp. CFR 303 (Murthy and Naidu 2010) was in acidic range i.e. 4.5–5.5. However, xylanase from *A. niveus* RS2 (Sudan and Bajaj 2007) showed pH optima of 7 while those from *A. tubingensis* and *A. terreus* showed optimum activity at pH 8 (Bakri et al. 2010). Similarly, *T. thermophilus* xylanase exhibited optimum activity at pH 7–8 (Maalej et al. 2009). In contrast, *P. citrinum* xylanase (Dutta et al. 2007) and two xylanases from *A. sydowii* SBS 45 (Nair et al. 2008) showed optimum activity at pH 8.5 and 10, respectively.

### Thermostability and pH stability of xylanase

**Fig. 8:**
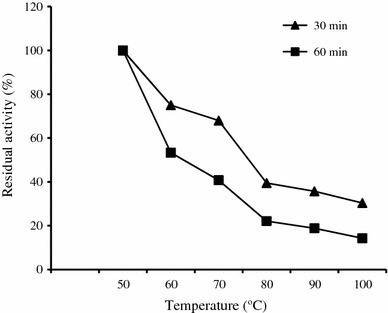
Thermostability of *A. fumigatus* MA28 xylanase. The enzyme (without substrate) was pre-incubated at different temperatures (50–100 °C) for varying time periods (30 and 60 min) and then assayed for residual activity. Initial activity was considered as 100%

*A.fumigatus* MA28 xylanase showed thorough stability at 50 °C (100%). At 60–70 °C, considerable high activity was observed after 30 min (68–75%) and 60 min (40–53%) of pre-incubation. However, at still higher temperatures (80–100 °C) the residual activity decreased significantly particularly after 60 min of incubation (residual activity: 14–21%) but up to 30 min the enzyme retained relatively higher activity (30–40%) as presented in the Fig. 8. The results indicate that the xylanase from *A.fumigatus* MA28 is moderately thermostable and may have potential for application in industrial processes. *A. foetidus* xylanase retained 71 and 20% of the activity after 30 min, at 50 and 60 °C, respectively, however, at 70 °C enzyme was completely inactivated within 30 min (Shah and Madamwar 2005). *Penicillium* sp. ECU0913 xylanase showed good stability for 2 days at 25 °C but after 1 week residual activity was 65% (Shi et al. 2011). Xylanase I and II from *A. sydowii* SBS 45 showed maximum stability at 30 °C for 4 h (Nair et al. 2008). Xylanases from *A. fumigatus* and *A. niveus* showed high stability at 60 °C for 30 min (95–98% of the initial activity), however, 60 min pre-incubation at this temperature caused considerable activity reduction (Peixoto-Nogueira et al. 2009).

**Fig. 9:**
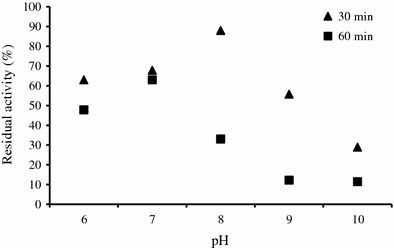
Stability of *A. fumigatus* MA28 xylanase at different pH. The enzyme (without substrate) was pre-incubated for 30 and 60 min at different pH using appropriate buffers: citrate buffer (pH 6), tris buffer (pH 7, 8 and 9) and glycine-NaOH (pH 10), and then assayed for residual activity. Initial activity was considered as 100%

pH stability analysis of xylanase showed that residual activity of xylanase after 30 min was substantial at pH 8 (88%), moderate at pH 9 (56%) and low at pH 10 (29%). In general, the enzyme was moderately stable up to 30 min at pH 6–9 as shown in Fig. 9. However, 60 min pre-incubation of enzyme at alkaline pH caused considerable activity reduction. In contrast, both xylanases I and II from *A. sydowii* SBS 45 showed broad pH (4–11) stability for 30 min-1 h (Nair et al. 2008). The pH stability of the xylanase from *A*. *fumigatus* was higher at pH 6.0–8.0, while the enzyme from *A*. *niveus* was more stable at pH 4.5–6.5 (Peixoto-Nogueira et al. 2009). Maximum stability of *R.* var. *microsporus* 595 xylanase was observed in the pH range of 5.0–6.0 (Shuvaeva and Sysoeva 2010).

### Effect of various additives on xylanase activity

**Fig. 10:**
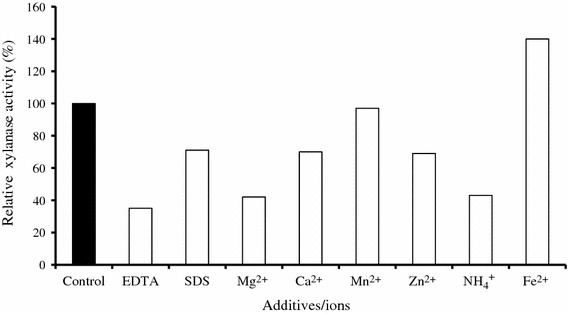
Effect of various additives and cations on xylanase activity. *Black bar* indicates activity without any additive/ion (control) while the *colourless bars* show xylanase activity when either of the additive or ion was included in enzyme assay mixture at final concentration of 10 mM

Various cations (Mg^2+^, Ca^2+^, Mn^2+^, Zn^2+^, NH_4_^+^, Fe^2+^) or additives (SDS and EDTA) were individually included in the assay mixture at final concentration of 10 mM, and activity assayed. Fe^2+^ enhanced the activity of xylanase by 40%. EDTA and Mg^2+^ moderately inhibited the xylanase and resulted in loss of activity by 65 and 58%, respectively (Fig. 10). All the other cations showed more or less inhibitory effect on xylanase activity. Negligible loss of activity (3%) was caused by Mn^2+^, while Ca^2+^ and Zn^2+^ decreased xylanase activity by 30 and 31%, respectively. SDS decreased enzyme activity by 29%. The decrease in enzyme activity may be due to involvement of cations/additives with the structure and conformation of the enzyme. Metals like Al^3+^, Ba^2+^, Ca^2+^, Na^+^ and Zn^2+^ enhanced the activity of xylanase I and II from *A. sydowii* SBS 45 at 10 mM concentration (Nair et al. 2008). All three purified iso-xylanases from *Myceliophthora* sp. IMI 387099 showed enhanced activity in presence of Na^+^, Mg^2+^ Mn^2+^ and K^+^ ions, whereas, Zn^2+^ and Cu^2+^ showed negative effect on Xyl IIa. The activity of Xyl IIa increased in presence of reducing agents dithiothreitol and mercaptoethanol, however, SDS showed inhibitory effect (Badhan et al. 2008). Activity of *A. niveus* RS2 was enhanced in the presence of Mn^2+^ ions while Hg^2+^ ions strongly inhibited the enzyme; other ions like Co^2+^, Ca^2+^, NH_4_^+^, Fe^2+^ and Mg^2+^ caused low to moderate activity reduction (Sudan and Bajaj 2007).

## Conclusion

The present study is a step-forward towards valorization of agro-residues for the production of value-added industrial products. Among various agro-residues examined wheat bran appears to be the most promising substrate for xylanase production by *A. fumigatus* MA28. Furthermore, the enzyme possessed substantial thermo-alkalistability and could be of potential significance for various industries including pulp and paper. Such enzymes must be studied in-depth for their structural conformation and encoding genes to decipher the molecular basis of thermo-alkalistability.
